# Deep phenotyping of infants and toddlers undergoing food oral immunotherapy (DINOSAUR): design and baseline characteristics of a prospective multi-omics cohort

**DOI:** 10.3389/falgy.2026.1919415

**Published:** 2026-07-28

**Authors:** Ann-Marie Malby Schoos, Frederikke Rosenvinge Skov, Sissel Vesterager Borge, Raymond Mak, Tiffany Wong, Stephanie C. Erdle, Ho Pan Sham, Bruce A. Vallance, Lianne Soller, Edmond S. Chan

**Affiliations:** 1 COPSAC, Copenhagen Prospective Studies on Asthma in Childhood, Herlev and Gentofte Hospital, University of Copenhagen, Copenhagen, Denmark; 2 Department of Pediatrics, Central and Western Zealand Hospital, Copenhagen University Hospital, Slagelse, Region Zealand, Denmark; 3 Department of Pediatrics, Copenhagen University Hospital - Amager and Hvidovre, Hvidovre, Denmark; 4 Department of Clinical Medicine, University of Copenhagen, Copenhagen, Denmark; 5 Division of Allergy, Department of Pediatrics, University of British Columbia, Vancouver, British Columbia, Canada; 6 Gut4Health Microbiome Profiling Core, BC Children's Hospital Research Institute, University of British Columbia, Vancouver, British Columbia, Canada; 7 Division of Gastroenterology, Hepatology and Nutrition, Department of Pediatrics, University of British Columbia, Vancouver, British Columbia, Canada

**Keywords:** biomarkers, food allergy, infants, microbiome, multi-omics, oral immunotherapy (OIT), precision medicine

## Abstract

**Background:**

The mechanisms influencing successful outcomes of oral Immunotherapy (OIT) are poorly understood. This study investigates the role of environmental, microbial, genetic, and immunological factors on the effectiveness of OIT in infants.

**Objectives:**

To identify biological and environmental mechanisms associated with successful OIT in infants by characterizing longitudinal changes in the microbiome and other multi-omic markers and relating these changes to clinical treatment outcomes.

**Methods:**

The Deep phenotypINg Of infantS And toddlers Undergoing food oral immunotheRapy (DINOSAUR) study is a prospective cohort study with two groups: infants (<18 months) undergoing OIT, and a comparison group (18–36 months) on the OIT waitlist. In the OIT group, biomaterial (blood, stool, urine, throat and skin swabs, skin tape strips, saliva, and nasal lining samples) and extensive information on environmental exposures has been collected at baseline immediately before initiation of OIT and ongoingly at the end of OIT (exit time-point), and has been collected once in the comparison group (age-matched to the exit time-point). Participant recruitment and sample collection were completed at BC Children's Hospital, Vancouver, Canada. Multi-omics analyses of the collected samples are ongoing and will be reported separately.The primary outcome is changes in the composition and functional profile of the oral- and gut microbiome during infant OIT.

**Results:**

A total of 81 participants with suspicion of food allergy attending the BC Children's Hospital Allergy Clinic between March 2023 and November 2023 were included; 58 infants in the OIT group and 23 children in the age-matched comparison group awaiting OIT. Of the 58 infants, 5 turned out not to have an IgE-mediated allergy. Their samples were archived and regarded as “non-food allergic controls”. In the infant group (*n* = 53), 66% were male, 85% had atopic dermatitis, and 42% were born by cesarean section. The majority (70%) were of Asian descent and lived in an urban environment (90%). The most common allergies were against peanut (51%), egg (40%) and cow's milk (26%).

**Conclusions:**

This prospective cohort study is the first of its kind to employ a systems biology approach to investigate how extensive biological and environmental factors influence OIT outcomes.

## Introduction

Food allergies affect approximately 4%–7% of children and can have severe, life-threatening consequences (1–3). Oral immunotherapy (OIT) is a promising approach to desensitize patients with food allergies, by gradually increasing the amounts of allergenic food they consume under medical supervision (4, 5). This process modulates the immune response and, in some cases, leads to sustained unresponsiveness, allowing patients to incorporate the food safely into their diet, although reported rates vary substantially across allergens and treatment protocols (6).

OIT induces several immunological changes aimed at reducing allergic sensitivity and promoting tolerance. The OIT process begins with early desensitization, characterized by decreased reactivity of effector cells and modulation of cytokine profiles. Over time, there is a reduction in allergen-specific IgE, an increase in IgG4, and expansion of regulatory T cells, all contributing to a more tolerogenic immune environment (7). For some individuals, these changes can lead to sustained unresponsiveness, potentially allowing for long-term tolerance even after OIT is discontinued.

OIT has shown particularly impressive safety outcomes in infants (6, 8), however, the effectiveness and safety of OIT vary significantly among individuals (9). Some children progress smoothly through the treatment, while others experience adverse reactions (allergic reactions including anaphylaxis), requiring treatment adjustments or discontinuation (4). Understanding the biological and environmental factors that contribute to these differences is crucial to optimizing OIT protocols and identifying children who are most likely to benefit.

Increasing evidence suggests that the gut and oral microbiomes play an important role in immune regulation and the development of allergic diseases. Several studies have demonstrated associations between early-life gut microbiome composition and the risk of food allergy development (10–14). For example, a murine study demonstrated that colonization with specific commensal bacterial communities from healthy infants can protect against food allergy by promoting regulatory immune responses (11). Similarly, prospective cohort studies have shown that distinct gut microbial profiles are associated with the development and resolution of milk and egg allergy in early childhood (13, 14), and one study of seven adults undergoing peanut OIT found increased microbial diversity and overrepresentation of several Clostridial species compared to baseline (15). Another recent study of 30 children undergoing OIT reported modest shifts in gut microbiota composition, with changes in beta diversity and partial normalization of specific bacterial phyla toward levels observed in non-allergic controls (16). However, these studies were limited to gut microbiota assessed by 16S rRNA sequencing and lacked detailed clinical phenotyping, longitudinal sampling across multiple biological sites, and integration with immunologic and environmental data. While these findings suggest a possible role of the microbiome in allergic disease, relatively little is known about how microbial communities influence responses to food immunotherapy. Additionally, the immunological, and metabolic changes that accompany OIT remain largely unexplored.

The Deep phenotypINg Of infantS And toddlers Undergoing food oral immunotheRapy (DINOSAUR) study addresses this knowledge gap by combining longitudinal microbiome profiling with detailed immunologic and environmental characterization during OIT. Previous studies examining the microbiome in food allergy have primarily focused on the gut microbiota alone. In contrast, DINOSAUR also investigates the oral microbiome, airway mucosal immune environment, and skin barrier biology. These compartments represent key interfaces between environmental exposures and the immune system and may therefore play important roles in shaping tolerance development. By integrating microbial, immunologic, genetic, environmental, and clinical data, the study aims to identify biological networks associated with successful OIT outcomes. Further, we compare infants before and after OIT, as well as a comparison group of children on the waitlist for OIT, to identify key predictors of OIT success and potential biomarkers that may support future precision medicine approaches, including individualized treatment selection, tailored updosing schedules, identification of children who may benefit from closer monitoring or adjunctive therapies, and improved prediction of treatment response and adverse reactions.

Based on previous studies, we hypothesize that: 1) OIT induces longitudinal changes in the gut and oral microbiome beyond those expected from normal maturation alone; 2) these microbial changes are associated with successful clinical response to OIT and accompanied by coordinated changes in immune and metabolic pathways; and 3) early-life environmental exposures and epithelial barrier function contribute to inter-individual variability in these biological responses and treatment outcomes.

## Objectives

The primary objective of the DINOSAUR study is to identify longitudinal biological changes during OIT, with particular emphasis on the gut and oral microbiome, and to determine how these changes relate to clinical treatment response. Specifically, we aim to:

- Assess longitudinal changes in the gut and oral microbiome during OIT.
- Determine whether these microbial changes are associated with successful, partial, or non-response to OIT.
- Investigate immune biomarkers and gene expression patterns associated with OIT response.
- Explore environmental factors associated with both microbiome changes and treatment success.

## Methods

### Participants

The DINOSAUR study is a prospective observational study of children diagnosed with food allergy and treated at BC Children's Hospital Allergy Clinic, Vancouver, Canada. Children were enrolled between March 2023 and November 2023 based on the following inclusion and exclusion criteria:

Inclusion criteria:

Infant group
  - Age 4–18 months AND either
  - A history of an allergic reaction to the food (home/OFC), and a positive skin prick test ≥ 3 mm or positive specific IgE ≥ 0.35 kUA/L, OR
  - No previous intake of the food but a specific IgE ≥ 5 kUA/L
  - About to start OIT
Comparison group
  - Age 18–36 months AND either
  - A history of an allergic reaction to the food (home/OFC) and a positive skin prick test ≥ 3 mm or positive specific IgE ≥ 0.35 kUA/L OR
  - No previous intake of the food but a specific IgE ≥ 5 kUA/L
  - On the waitlist for OIT

Exclusion criteria:

- Uncontrolled asthma
- Severe atopic dermatitis requiring systemic therapy
- Uncontrolled eosinophilic esophagitis
- Ongoing OIT at time of inclusion (including egg- and milk ladders)

Among the infants, five turned out not to have an IgE-mediated food allergy after the baseline samples were collected, and we will show their results as “non-food allergic controls” – i.e., children without food allergy.

### Intervention

**Figure 1:**
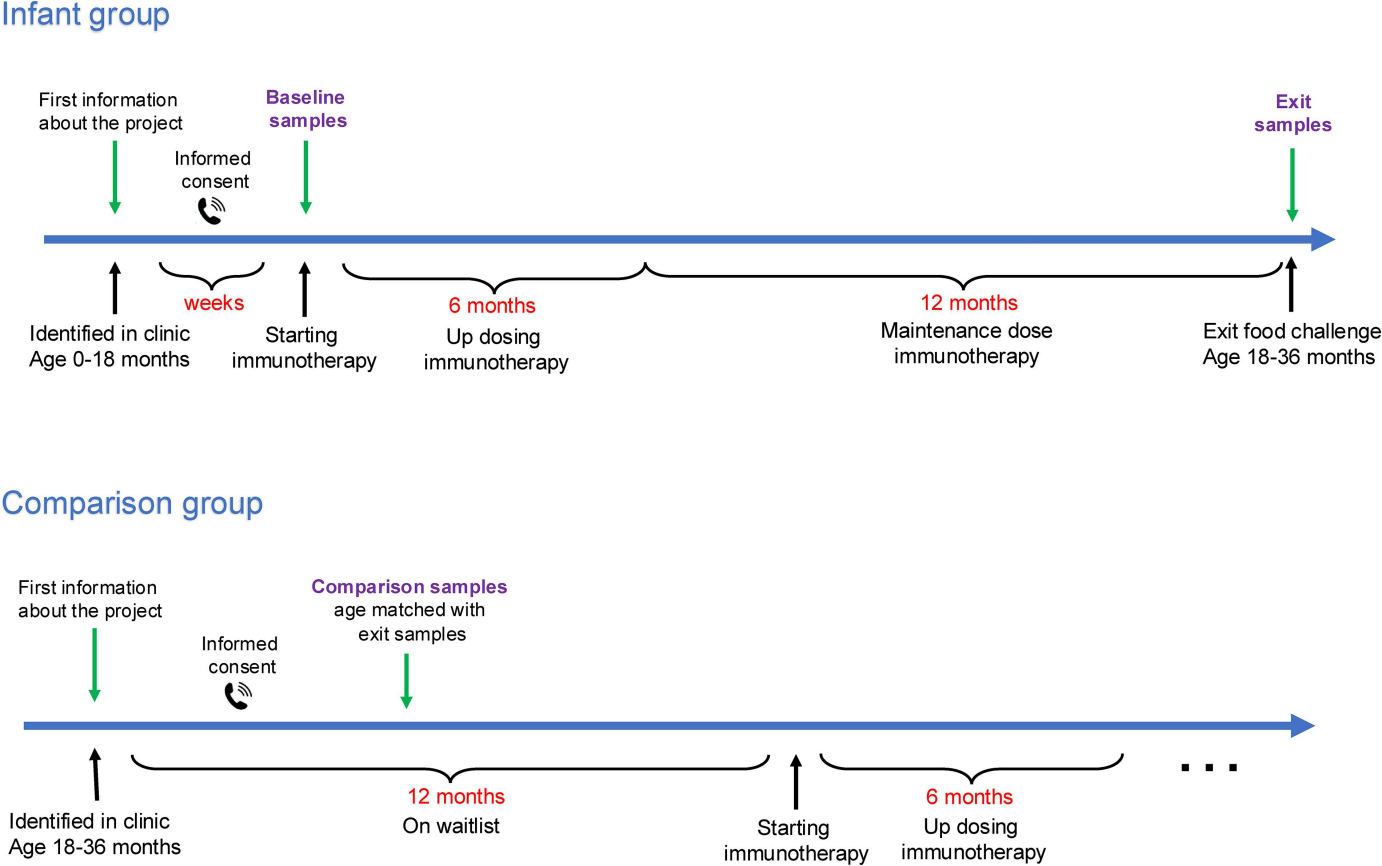
Study design of the DINOSAUR cohort. Overview of the prospective cohort design including the infant oral immunotherapy (OIT) group and the age-matched comparison group. Infants (<18 months) undergoing OIT were sampled at baseline immediately before initiation of OIT between March and November 2023 and after approximately 18 months at the exit oral food challenge (ongoing due to delays). The comparison group (18–36 months) provided a single cross-sectional sample collected between March and November 2023 during the waiting period for OIT.

All the children in the infant group were enrolled in the clinic's OIT program (4, 17–19) where they ingested increasing amounts of allergenic food(s) over approximately 6 months (updosing), and then maintaining the target dose (300 mg protein) for 1 year. At the end, the children performed an exit OFC with a full serving of the allergenic food(s) (Figure 1). OIT was initiated for the clinically relevant food allergen(s) based on each participant's clinical history, allergy testing, and physician assessment. Participants could undergo OIT for up to five food allergens simultaneously when clinically indicated.

The children in the infant group had their first round of samples collected at baseline (“baseline samples”) at age <18 months, and the second round (“exit samples”) after approximately 18 months of OIT (at their exit OFC visit; age 18–36 months, still ongoing due to delays). The children in the comparison group (age 18–36 months) had one round of samples collected (“comparison samples”) during the waiting period for OIT and were used as an aged-matched comparison group for results of the infant OIT exit samples (see Figure 1).

The following definitions will be used to define the outcome of the exit OFC (ongoing) (17):

**Successful responders** - children who complete OIT updosing and maintenance without complications and pass a 4,000 mg protein exit OFC.
**Partial responders** - children with difficulties updosing due to allergic reactions, and/or cannot tolerate 4,000 mg protein at the exit OFC, but who increased their threshold of reaction to at least 1,000 mg protein.
**Non-responders** - children who drop out of OIT due to allergic reactions, and/or have a threshold <1,000 mg protein at the exit OFC.

### Data collection and management

**Figure 2:**
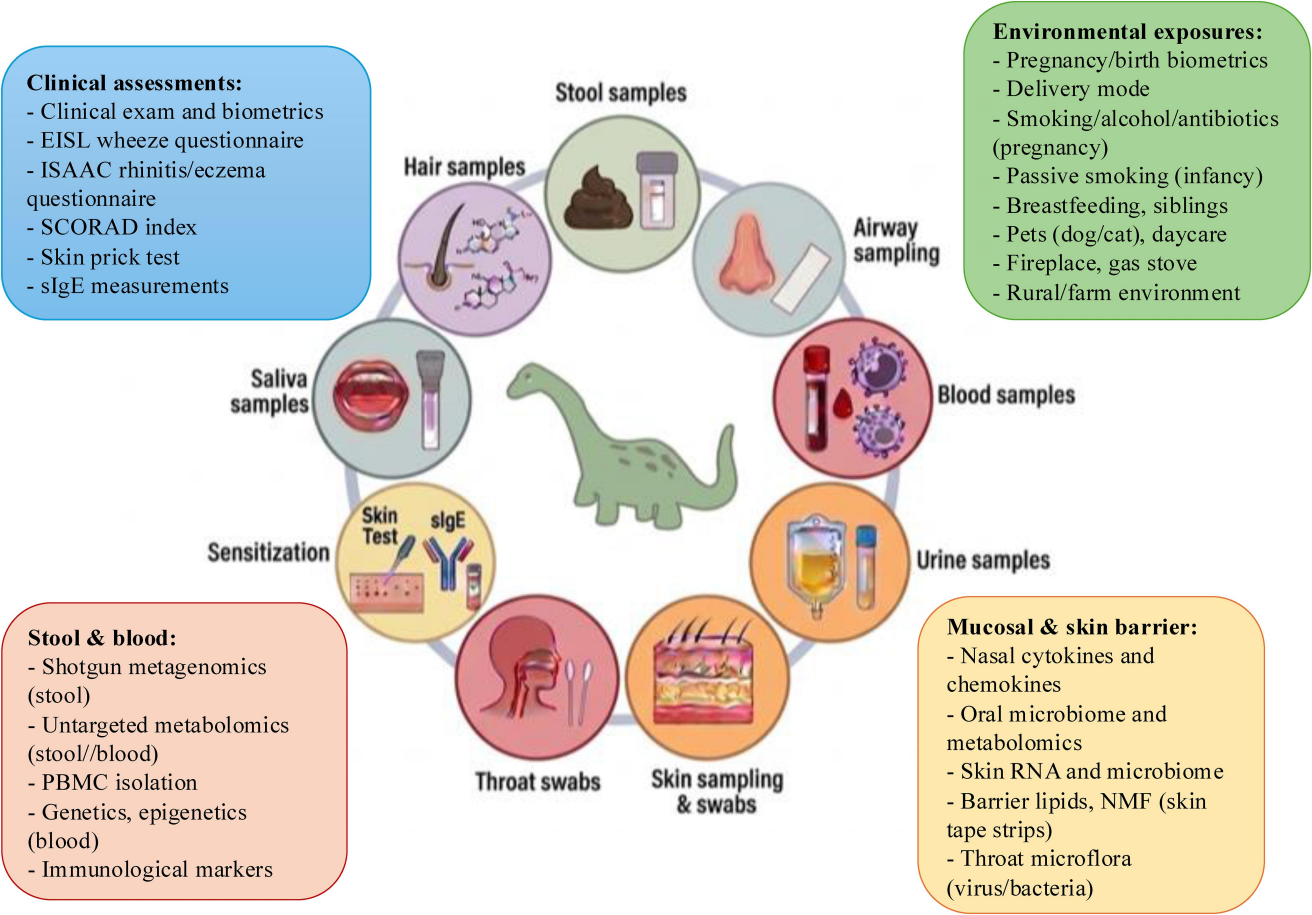
Overview of clinical data and multi-omics sampling in the DINOSAUR study. Schematic illustration of data collection and biological sampling across study visits, including clinical phenotyping, environmental exposures, and multi-omics profiling (microbiome, metabolomics, immunologic, genetic, and transcriptomic data) from multiple biological compartments.

The following data will be investigated and constitute important components for endotyping the children, thus exploring predictive markers of who will respond to the OIT. Further, by analyzing changes in these factors during OIT, they can help explain some of the mechanisms behind OIT. All the collected data are shown in Figure 2.

#### Clinical examinations and interview

We did a clinical examination of the children and recorded biometrics during all visits. The parents were interviewed about the home environment including passive smoking, pets in the home, fireplace, gas stove, breastfeeding (exclusive and partial), siblings, living environment (rural or farm), daycare attendance, health status of the child, exposure to antibiotics, introduction to allergenic foods, and socioeconomic status. Further, information on pregnancy exposures (smoking/passive smoking, alcohol, and antibiotics), delivery mode, birth biometrics, and gestational age was collected. When a child had current symptoms of wheeze, allergic rhinitis and/or atopic dermatitis, we expanded the interview to include symptom severity and frequency (International Study of Wheezing in Infants [EISL] questionnaire (20) and International Study on Asthma and Allergies in Childhood [ISAAC] questionnaires (21)). The Hanifin and Rajka criteria (22) were used to elaborate the diagnosis of atopic dermatitis and the severity was scored using the SCORing Atopic Dermatitis (SCORAD) index (23).

#### Stool samples

Stool samples were collected by the participants either during the in-person visit or in their own home and sent directly to the Gut4Health Microbiome Core Facility (RRID: SCR_023673), where they were stored at −80 °C. The stool samples will be analyzed using shotgun metagenomic sequencing and untargeted metabolomics.

#### Blood samples

Blood samples were drawn at the time of the visits and transferred directly to BC Children's Hospital BioBank for separation of blood cells and plasma and stored at −80 °C. Isolation of peripheral blood mononuclear cells (PBMCs) was done by the lab technician and the PBMCs were stored in liquid nitrogen. The blood samples will be analyzed for immunological, genetic, epigenetic, and metabolomic markers. The PBMCs will be used for future immunological and transcriptomic analyses aimed at identifying cellular mechanisms associated with treatment response.

#### Hair sample

A hair sample (1 cm/ 2 grams) was collected and will be analyzed for cotinine and cortisol, which are markers of tobacco smoke exposure and stress.

#### Airway mucosal immune profile

Nasal lining fluids were sampled from the upper airways using a non-sterile, single-use device consisting of a synthetic absorptive matrix (SAM) strip attached to an applicator handle housed in a cryo-transport tube for protection (NASOSORPTION^TM^, Hunt Developments Ltd.). The SAM was inserted gently by means of direct vision into the nasal cavity laterally against the anterior portion of the inferior turbinate to absorb nasal secretion. The samples were stored directly at −80 °C for later analysis of immunological signaling molecules.

#### Saliva samples

Saliva samples were collected from the vestibular area of the mouth using the Micro•SAL^TM^ Infant Saliva Collection Device which is intended for standardized collection of saliva from infants. The saliva samples were stored directly at −80 °C for later analysis of the oral microbiome composition, using shotgun metagenomic sequencing and untargeted metabolomics.

#### Tape stripping

Fifteen consecutive stratum corneum tape strips were collected from non-lesional skin on the volar forearm. Circular adhesive tapes (D100 - D-Squame Standard Sampling Discs, diameter: 22.0 mm) were placed at the same skin site and pressed for ten seconds with the D500 - D-squame Pressure Instrument. The six deepest tape strips were placed directly into Qiagen RLT buffer and stored at −80 degrees for later analysis of microbiome DNA, natural moisturizing factor, proteomics, lipidomics, and transcriptomics (deepest layers).

#### Throat swabs

Two throat swabs (one for virus analysis and one for bacterial analysis) were simultaneously inserted in the mouth to swab the palate. The swabs were stored directly at −80 °C for later analysis of the microflora of the oral cavity and the upper airways.

#### Skin swabs

Skin swabs were collected using sterile swabs pre-moistened with a few drops of sterile saline before sampling at the volar side of the forearm and the armpit to investigate the skin microflora. The swabs were stored directly at at −80 °C for later analysis of the skin microbiome.

#### Urine sample

Urine samples were collected either during the visit or in their own home and sent directly to BC Children's Hospital BioBank, where they were stored at −80 °C for later analysis of immunologic and metabolic compounds. In children who were not toilet-trained, urine was collected using a pediatric urine collection bag.

#### Measurements of sensitization

At each visit, the allergist determined which foods to evaluate using skin prick testing and specific IgE (sIgE), generally limited to foods with a suspected clinical reaction. sIgE levels were measured using ImmunoCAP (ThermoFisher Scientific), and skin prick tests were performed with standard allergen extracts (ALK Abelló and Stallergenes Greer). Histamine dihydrochloride (10 mg/mL) and physiological saline (9 mg/mL) served as positive and negative controls, respectively. Antihistamine usage was not allowed 72 h before skin prick testing (the positive control was used as screening for use of antihistamines with longer half-life), nor were mild steroid creams (group 1–2) on the arms 24 h before testing, or stronger steroid creams (group 3–4) 14 days before testing.

### Outcomes

The primary mechanistic outcome of the DINOSAUR study is longitudinal change in the composition and functional profile of the oral and gut microbiome during infant OIT. We will analyze the diversity and function (metabolomics) of the microbiota including abundance of specific species and how they change during immunotherapy (baseline vs. exit sample). Specific attention will be paid to microbes with known immunomodulatory properties, including those producing short-chain fatty acids or aryl hydrocarbon receptor ligands (e.g., Clostridial and Lactobacillus species) (15, 24, 25). The exit sample will also be compared to the comparison sample to ensure that the microbiome composition differs between the two groups, and that the changes observed is not simply due to age-dependent maturation of the gut (exit vs. comparison samples). The primary clinical outcome is OIT response at the exit OFC, classified as successful, partial, or non-response. The central hypothesis is that microbiome changes occurring during OIT are associated with, and may predict or help explain, differences in clinical treatment response.

Secondary analyses will examine changes in immune biomarkers, gene expression, metabolomics, and environmental exposures in relation to both microbiome trajectories and clinical outcomes.

### Risks and discomfort

Most study procedures are part of routine clinical care, with minimal additional risks. Blood draws and skin tape stripping may cause mild discomfort. Collecting stool, saliva, and airway samples are non-invasive and present minimal risk

### Safety and adverse events

All participants undergo routine clinical monitoring. Adverse events related to OIT (e.g., allergic reactions) are documented and managed according to standard clinical protocols. Participants can withdraw from the study at any time

### Ethics

This study is approved by the UBC BC Children's Hospital Ethics Committee (H22-00997). Participants' parents have all provided informed consent. Participant confidentiality is maintained through de-identified data storage and restricted access protocols.

### Statistical analysis plan

Multi-Omic Data Integration will be performed using Sparse Canonical Correlation Analysis (sCCA) implemented via the PMA package in R. Prior to analysis, datasets will be log-transformed, standardized, and filtered for high-variance features, with missing data imputed using k-nearest neighbors. sCCA will be applied pairwise between clinical data, immunological, metagenomics, and metabolomics datasets to identify correlated features across omic layers.

All the baseline data will be analyzed using a systems biology approach combining the clinical, immunological, and molecular data to explore if any of these markers help predict the exit OFC outcomes including unwanted side effects.

In all analyses, we will consider sex as a confounder and other possibly relevant confounders from the exposome (antibiotic use, cesarian section etc.) will also be included in the analyses.

## Results

### Demographics

We recruited 81 children in this study of whom 58 were in the infant group, but 5 of the infants turned out to not have food allergies and were hence considered non-food allergic controls in the analysis. This left 53 infants and 23 comparisons. The median age in the infant group was 12 months [interquartile range (IQR) 10–14 months]; 66% were male and 70% had one or both parents with an Asian (South- and East Asian combined) ethnic background (Table 1). A total of 19% had started daycare and 33% had an older sibling. The majority of families were living in an urban environment (90%).

**Table 1:** Baseline characteristics of infants undergoing oral immunotherapy, comparison participants, and non-food allergic controls.

Sample Type	Infant group (at baseline) *N* = 53	Comparison group *N* = 23	*p* ^b^	Non-food allergic controls *N* = 5
Demographics				
Male sex	35 (66%)	14 (61%)	0.67	5 (100%)
Age, months	12.0 (10.0–14.0)	34.0 (31.2–38.8)	**<0.001**	9.0 (9.0–11.0)
Ethnic background^a^				
White	19 (36%)	6 (26%)	0.85	2 (40%)
Hispanic	3 (5.7%)	1 (4.3%)		0
Asian	37 (70%)	17 (74%)		4 (80%)
Black	2 (3.8%)	1 (4.3%)		0
Other	2 (3.8%)	0		2 (40%)
Birth information				
Delivery mode				
Vaginal	30 (58%)	13 (57%)	0.92	4 (80%)
C-section	22 (42%)	10 (43%)		1 (20%)
Season of birth				
Winter	15 (29%)	7 (30%)	0.26	1 (20%)
Spring	7 (13%)	4 (17%)		1 (20%)
Summer	18 (35%)	4 (17%)		2 (40%)
Fall	12 (23%)	8 (35%)		1 (20%)
Socioeconomic status				
Maternal age at birth, years	33.1 ± 4.3	33.6 ± 5.6	0.72	35.80 ± 3.35
Paternal age at birth, years	34.4 ± 5.3	34.8 ± 5.8	0.36	38.00 ± 4.42
Maternal education				
Min. college	15 (29%)	4 (18%)	0.17	1 (20%)
Bachelor	20 (38%)	12 (55%)		3 (60%)
University	17 (33%)	6 (27%)		1 (20%)
Paternal education				
Min. college	18 (35%)	7 (33%)	0.94	1 (20%)
Bachelor	19 (37%)	8 (38%)		3 (60%)
University	15 (29%)	6 (29%)		1 (20%)
Household income (CAD)				
<80,000	8 (16%)	2 (9.0%)	0.80	0
80,000–120,000	15 (29%)	7 (32%)		1 (20%)
120,000–160,000	8 (16%)	4 (18%)		1 (20%)
>160,000	20 (39%)	9 (41%)		3 (60%)
Parental disposition				
Maternal asthma	10 (19%)	4 (17%)	0.84	1 (20%)
Paternal asthma	16 (31%)	4 (18%)	0.28	1 (20%)
Maternal atopic dermatitis	19 (37%)	7 (30%)	0.58	2 (40%)
Paternal atopic dermatitis	17 (33%)	5 (23%)	0.42	1 (20%)
Maternal food allergy	8 (15%)	2 (8.7%)	0.50	0
Paternal food allergy	9 (17%)	1 (4.5%)	0.17	0
Maternal allergic rhinitis	20 (38%)	9 (39%)	0.94	1 (20%)
Paternal allergic rhinitis	33 (63%)	11 (48%)	0.21	2 (40%)
Atopic diseases				
Wheeze child	7 (13%)	5 (22%)	0.33	0
Atopic dermatitis child	44 (85%)	16 (70%)	0.18	2 (40%)
Allergic rhinitis child	8 (15%)	9 (39%)	**0.03**	1 (20%)

a Can be more than one.

b Infants vs. comparison using Welch's *t*-test for continuous variables and *χ*² test or Fisher's exact test for categorical values.

Bold values indicate a statistically significant difference.

### Pregnancy and birth

The children in the infant group were overall healthy babies born at term with a mean birth weight of 3,396 g, and a mean Appearance, Pulse, Grimace, Activity, and Respiration (APGAR) score of 10 at 5 min. The majority were born during summertime (35%), and 42% were born by cesarean section (Table 1).

### Early exposures

In the infant group, a total of 71% children had been exposed to dogs in their first year of life (25% > 100 days) and 33% had been exposed to cats (9.6% > 100 days). Very few children had been exposed to smoking during pregnancy or passive smoking during infancy (3.8% for both measures). Similarly, very few had been exposed to alcohol during pregnancy (more than 10 units total, 5.8%), antibiotics during pregnancy (15%), and antibiotics during infancy (12%). The mean duration of exclusive breastfeeding was 4.0 months (SD 2.5) and 60% of the children were still partially breastfed at the baseline visit.

### Socioeconomic status

In the infant group, the parents were generally highly educated and of relatively high socioeconomic status. The mean maternal age at birth was 33.1 years (SD 4.3) and the mean paternal age was 35.2 years (SD 4.9). Among mothers, 29% had completed at least college education, and similarly among fathers, 35% had completed at least college education. The total household income was relatively high, with 39% reporting an annual income above CAD 160,000 (Table 1).

### Atopic predisposition and current atopic disease

Among the infants, 92% had a parent with an atopic disease. The majority of the parents suffered from allergic rhinitis (63% of fathers and 38% of mothers). Most of the infants suffered from atopic dermatitis (85%) with a mean SCORAD of 20 during the baseline visit. Few had a history of wheeze (13%) and some had started having symptoms of allergic rhinitis (15%) (Table 1).

As per the inclusion criteria, the median age was higher in the comparison group (34 months vs. 12 months, *p* < 0.001). Further, there were more children with allergic rhinitis in the comparison group (39% vs. 15%, *p* = 0.03). The two groups were otherwise similar on all parameters (Table 1).

### Current food allergies

**Figure 3:**
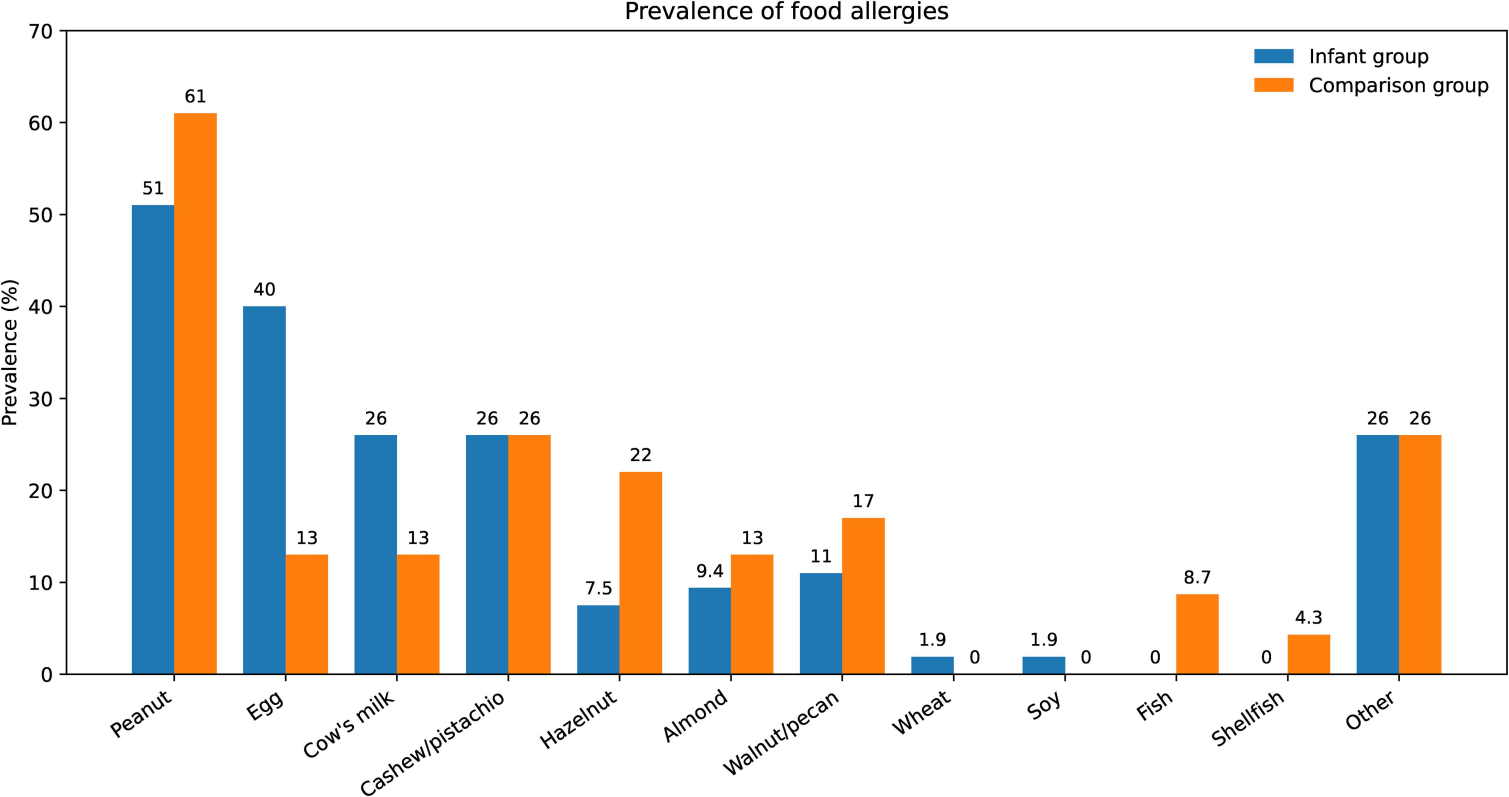
Bar chart illustrating the prevalence (%) of reported food allergies among children in the infant group (*n* = 53) and the comparison group (*n* = 23). Values above the bars indicate the percentage of children with each allergy.

The most common allergies in the study were against peanut (51% in infant group, 61% in comparison group), followed by egg and cow's milk (40% and 26%) in the infant group. As expected, the prevalence of egg and cow's milk allergy were lower in the comparison group, as those children were older (13% for both). After peanut, the comparison group was dominated by allergies to tree nuts, in particular cashew/pistachio (26%) (Figure 3).

### Sensitization levels in the infant and comparison groups

**Figure 4:**
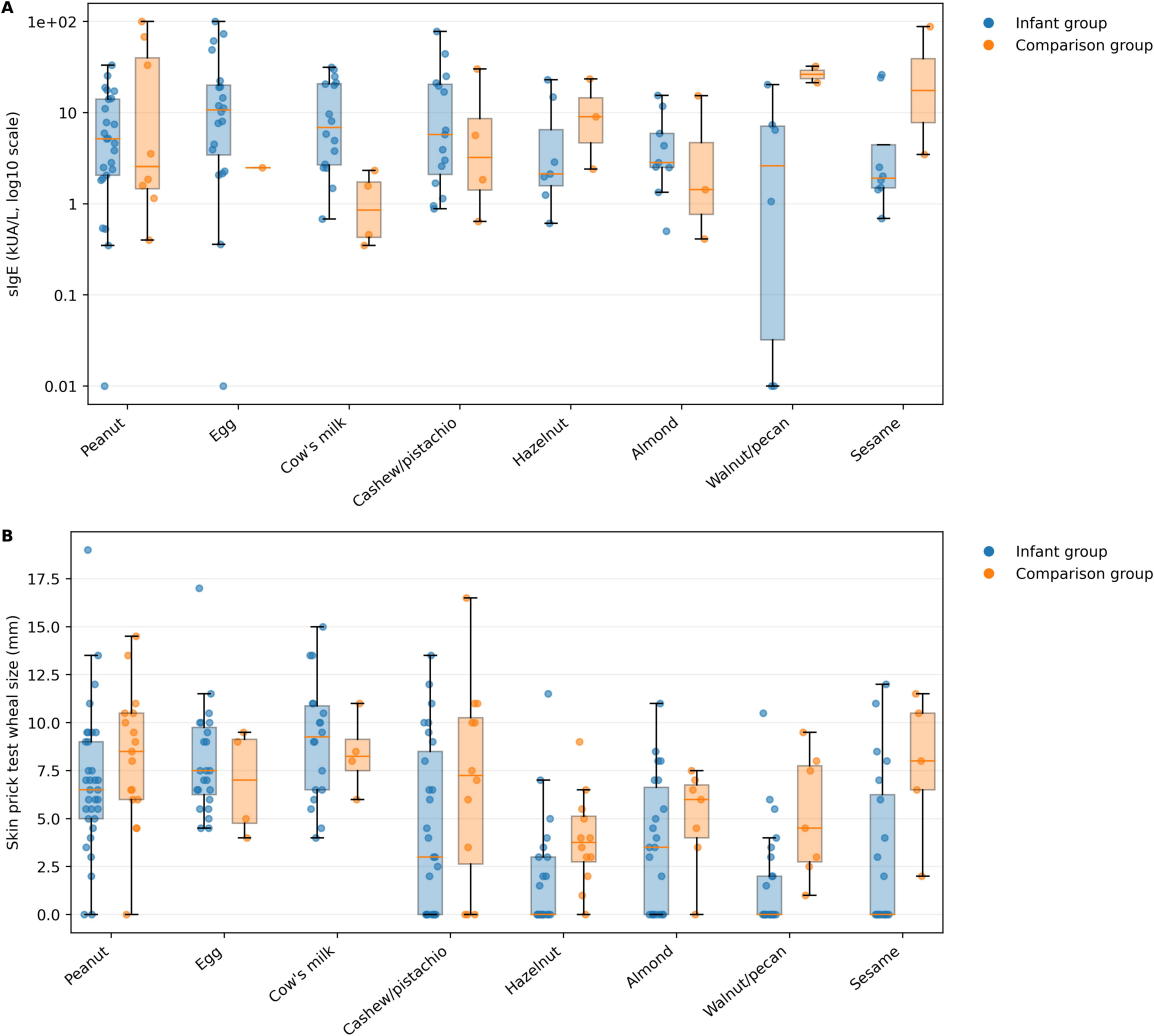
Scatter-box plots showing food-specific IgE (sIgE) levels for foods (+0.01kU_A_/L) **(A)** and skin prick test wheal sizes **(B)** in the infant group (*n* = 53) and the comparison group (*n* = 23). Boxes indicate the interquartile range with the median, and whiskers represent the distribution of values. In **(A)**, the *y*-axis is displayed on a log10 scale, with tick labels expressed as kUA/L for ease of interpretation. In **(B)**, wheal sizes are presented in millimeters (mm).

The sIgE and skin prick tests levels for the most common allergens in the infant and comparison groups are shown in Figure 4. sIgE levels and skin prick test wheal sizes varied widely across participants and allergens in both groups.

## Discussion

The DINOSAUR study builds upon the limited existing literature by providing a uniquely comprehensive systems biology framework for investigating mechanisms underlying OIT response. This baseline report describes the demographic, clinical, and sensitization characteristics of the enrolled cohort and outline the extensive multi-omic data collection that will enable systems-level investigation of mechanisms underlying OIT responses. The cohort is characterized by a high prevalence of atopic dermatitis, strong parental atopic history, and sensitization predominantly to peanut, egg, and cow's milk, reflecting the clinical population typically referred for early-life food allergy management.

OIT is an increasingly used therapeutic strategy for food allergy, with numerous studies demonstrating that many children can achieve desensitization through gradual allergen exposure. Meta-analyses of randomized trials have shown that OIT increases the threshold of reactivity for peanut, egg, and cow's milk allergy, although treatment responses remain heterogeneous and adverse reactions are common in some patients. Real-world studies have further demonstrated that preschool OIT can be both effective and safe when implemented in clinical practice, particularly in younger children (4, 17, 18). However, despite these promising outcomes, a substantial proportion of children experience difficulties during updosing or they fail to achieve full desensitization, highlighting the need to better understand the biological mechanisms underlying treatment variability.

An important consideration when interpreting longitudinal microbiome changes during OIT is that participants progressively increase their intake of the allergenic food as part of the treatment protocol. Consequently, observed alterations in microbial composition or function may reflect changes in dietary exposure in addition to immunologic mechanisms associated with tolerance induction (26). These processes are likely to be closely intertwined, as dietary substrates directly influence microbial ecology and metabolism while simultaneously driving immunologic adaptation through OIT (26). Rather than attempting to separate these effects completely, the DINOSAUR study aims to characterize the integrated biological response to OIT by combining longitudinal microbiome profiling with immune, metabolic, and clinical phenotyping. Furthermore, the inclusion of an age-matched comparison group of food-allergic children awaiting OIT helps distinguish treatment-associated changes from normal age-related microbiome maturation, although residual effects of dietary differences cannot be entirely excluded. Finally, the integration of shotgun metagenomics with untargeted metabolomics may help distinguish microbial compositional changes from alterations in microbial metabolic activity, providing additional insight into the biological pathways associated with OIT.

An additional strength of the DINOSAUR study is the inclusion of detailed characterization of skin barrier biology. Increasing evidence suggests that impaired skin barrier function is central to the pathogenesis of food allergy by facilitating epicutaneous allergen sensitization, promoting type 2 immune activation, and altering interactions between the host and skin microbiota (27). Beyond its established role in food allergy development, persistent skin barrier dysfunction may influence the induction of immune tolerance during OIT by maintaining epithelial type 2 inflammation and continued allergen exposure through the skin (27, 28). By integrating tape-strip-derived proteomic, transcriptomic, lipidomic, natural moisturizing factor, and skin microbiome data with longitudinal immune and microbiome profiling, the DINOSAUR study will enable exploration of whether skin barrier integrity represents a mechanistic determinant of OIT response. Although these mechanisms remain incompletely understood, identifying skin barrier signatures associated with treatment success may provide novel targets for patient stratification and future adjunctive therapies. Given the high prevalence of atopic dermatitis in the cohort (85%), investigating the contribution of skin barrier dysfunction to OIT response is particularly relevant and may help explain part of the observed heterogeneity in treatment outcomes.

Another important feature of the study design is the inclusion of an age-matched comparison group of children with food allergy who are awaiting OIT. Early childhood is characterized by rapid maturation of both the immune system and the microbiome, making it challenging to distinguish treatment-related changes from normal developmental trajectories. Previous microbiome studies in early life have demonstrated substantial age-related shifts in microbial composition during the first years of life (29, 30). By comparing exit samples from infants who have completed OIT with samples obtained from older children on the waitlist, the DINOSAUR study seeks to differentiate biological changes associated with OIT from those reflecting natural maturation.

The baseline characteristics of the cohort are consistent with known epidemiologic patterns of pediatric food allergy. Peanut allergy was the most common allergy in both groups, followed by egg and cow's milk allergy among infants. In the comparison group, which consisted of older children, egg and cow's milk allergies were less prevalent, whereas tree nut allergies were more frequent. This pattern is consistent with the natural history of food allergy, where milk and egg allergies often resolve during early childhood while peanut and tree nut allergies tend to persist longer (1, 2). Sensitization levels measured by specific IgE and skin prick testing varied widely across individuals, reflecting the clinical heterogeneity that may influence OIT outcomes.

The study also incorporates detailed environmental exposure data, including breastfeeding duration, antibiotic exposure, pet ownership, and other early-life factors that may influence immune development. Environmental exposures during infancy have been associated with both microbiome composition and allergic disease risk in several previous studies (10, 29, 30). Integrating these exposures with multi-omic biological data will allow exploration of complex interactions between environmental factors, microbial communities, and immune responses during OIT.

We observed a relatively high proportion (70%) of children with at least one parent of Asian ancestry in our cohort. In comparison, population-level data from British Columbia indicate that 18.6% of children under 15 years have an Asian background (including both single and multiple ethnic origin responses, comparable to our classification) (31). This suggests that children of Asian ancestry are overrepresented in our study population.

Several limitations should be considered when interpreting these baseline findings. First, the cohort size is relatively modest, which may limit statistical power for detecting associations within high-dimensional multi-omic datasets. However, the extensive biological sampling and longitudinal design provide a rich dataset for hypothesis generation and mechanistic exploration. Second, the study population is drawn from a single specialized allergy clinic, and the relatively high proportion of families of Asian ethnic background and higher socioeconomic status may limit generalizability to other populations. The high proportion of Asian families may however, provide unique insights into atopy among immigrant populations in Canada, potentially analogous to previously published findings from other countries (32). Finally, although the inclusion of a comparison group helps address age-related biological changes, the group was sampled cross-sectionally rather than longitudinally, and therefore seasonal and other temporal environmental factors cannot be completely separated from treatment-related effects. Also, some residual confounding related to age differences between groups cannot be excluded.

Despite these limitations, the DINOSAUR study represents one of the most comprehensive efforts to investigate biological determinants of OIT responses in early childhood, and the first study to examine gut and oral microbiome and metabolome outcomes during OIT.

From a clinical perspective, the immediate goal of the DINOSAUR study is not to support routine multi-omics profiling before OIT, but to identify biological signatures that may improve understanding of treatment heterogeneity. In the future, such biomarkers could contribute to precision medicine approaches by identifying children who are most likely to respond successfully to OIT, those at increased risk of adverse reactions or difficult up-dosing, and potentially those who may benefit from adjunctive therapies. Ultimately, these insights may support more individualized OIT protocols that improve both efficacy and safety.

In conclusion, the DINOSAUR cohort provides a unique platform for studying the biological mechanisms underlying OIT responses in infants and toddlers with food allergy. The integration of microbiome, immunologic, genetic, and environmental data may advance understanding of tolerance development and support the development of precision medicine strategies in pediatric food allergy management.
